# Differences in the Cellular Response to Acute Spinal Cord Injury between Developing and Mature Rats Highlights the Potential Significance of the Inflammatory Response

**DOI:** 10.3389/fncel.2016.00310

**Published:** 2017-01-13

**Authors:** Theresa C. Sutherland, Kathryn J. Mathews, Yilin Mao, Tara Nguyen, Catherine A. Gorrie

**Affiliations:** ^1^Neural Injury Research Unit, School of Medical and Molecular Bioscience, University of Technology SydneyUltimo, NSW, Australia; ^2^Discipline of Biomedical Sciences and Brain and Mind Centre, Sydney Medical School, The University of SydneySydney, NSW, Australia

**Keywords:** spinal cord injury, inflammation, neonates, age-related, microglia, endogenous neural progenitor cells, innate immune cells

## Abstract

There exists a trend for a better functional recovery from spinal cord injury (SCI) in younger patients compared to adults, which is also reported for animal studies; however, the reasons for this are yet to be elucidated. The post injury tissue microenvironment is a complex milieu of cells and signals that interact on multiple levels. Inflammation has been shown to play a significant role in this post injury microenvironment. Endogenous neural progenitor cells (NPC), in the ependymal layer of the central canal, have also been shown to respond and migrate to the lesion site. This study used a mild contusion injury model to compare adult (9 week), juvenile (5 week) and infant (P7) Sprague-Dawley rats at 24 h, 1, 2, and 6 weeks post-injury (*n* = 108). The innate cells of the inflammatory response were examined using counts of ED1/IBA1 labeled cells. This found a decreased inflammatory response in the infants, compared to the adult and juvenile animals, demonstrated by a decreased neutrophil infiltration and macrophage and microglial activation at all 4 time points. Two other prominent cellular contributors to the post-injury microenvironment, the reactive astrocytes, which eventually form the glial scar, and the NPC were quantitated using GFAP and Nestin immunohistochemistry. After SCI in all 3 ages there was an obvious increase in Nestin staining in the ependymal layer, with long basal processes extending into the parenchyma. This was consistent between age groups early post injury then deviated at 2 weeks. The GFAP results also showed stark differences between the mature and infant animals. These results point to significant differences in the inflammatory response between infants and adults that may contribute to the better recovery indicated by other researchers, as well as differences in the overall injury progression and cellular responses. This may have important consequences if we are able to mirror and manipulate this response in patients of all ages; however much greater exploration in this area is required.

## Introduction

Spinal cord injury (SCI) is a complex and evolving pathology that arises from an insult to the spinal cord; either a mechanical trauma (TSCI) or from a variety of non-traumatic causes. No matter the cause, spinal cord injury will result in some loss of motor and sensory function below the lesion site (Barnabé-Heider and Frisén, 2008) as well as some degree of autonomic dysfunction (Karlsson, 2006). There are as many manifestations of SCI as there are spinal cord patients; however, they all share a common basic pathophysiology (Kwon et al., 2004; Profyris et al., 2004; Rowland et al., 2008). This pathology is characterized by cell death and inflammation (Kwon et al., 2004; Norenberg et al., 2004), myelopathy, breach of the blood-brain barrier, and damage to both the glia and neural fiber tracts (Ronaghi et al., 2010). This leads to the disruption of nerve tracts and the loss of both motor neurons and interneurons, which in turn leads to the observed loss of function (Ronaghi et al., 2010).

SCI in immature cords is less well characterized as pediatric SCI is more rare, accounting for only 1–13% of all SCI (Apple et al., 1995; Lee et al., 2009). Clinically childhood SCI presents with different signs to those observed in teenage and adults patients with comparable injuries (Pape, 2012). This is due to distinct differences between mature and developing spinal cords, and consequently differences in the timeframe of development and the severity of the injury (Bilston and Brown, 2007; Furlan et al., 2010; Kuluz et al., 2010). Currently there is an accepted trend of higher initial severity in children than adults, although most support the notion that young survivors of the initial SCI have typically better and more rapid functional recoveries (Brown et al., 2005; Clarke and Bilston, 2008; Clarke et al., 2009; Furlan et al., 2010; Kuluz et al., 2010; Pape, 2012). Bregman and Goldberger (1983a) coined the term “infant lesion effect” to describe the observation that when spinal neural pathways are damaged in newborns the animal's motor function as an adult is superior to those undergoing the same lesion in adulthood (Kuluz et al., 2010). It has also been noted that the profile of the secondary damage phase could also vary in an age-dependant manner (Yuan et al., 2013).

It is widely accepted that the pathophysiology of SCI consists of two distinct components; (Kwon et al., 2004; Donnelly and Popovich, 2008; Rowland et al., 2008; Ross and Pawlina, 2011) the initial mechanical trauma and the developing secondary insult. The primary injury refers to the trauma that directly imparts a detrimental force on the spinal cord and results in the disruption of axons, the surrounding glial cells and blood vessels (Profyris et al., 2004). The secondary injury is delayed and manifests in a broad spectrum of pathologies that exacerbate the injury (Profyris et al., 2004; Rowland et al., 2008). This phenomenon is common to SCI in both adult and developing cords.

One of the important element of this pathophysiology is the inflammatory response. The body's own immune repose plays an important role in the progression of SCI (Chan, 2008), however it is still contentious whether this cascading immune response is beneficial or detrimental to recovery (Fleming et al., 2006; Donnelly and Popovich, 2008; Rowland et al., 2008). In a typical response to CNS trauma the first cells to arrive are neutrophils, within a matter of hours post injury, as well as exogenous macrophages and endogenous microglial activation (Fitch and Silver, 2008). Many elements of the inflammatory response have both neuroprotective and neurotoxic effects; which of these predominates is likely due to the timeframe and scale of expression, and the cells on which they are acting (Kwon et al., 2004). During the inflammatory cascade that occurs post injury in the CNS microglia and astrocytes are activated and neutrophils are recruited in the initial 24 h. Blood monocytes migrate into the injury site over the course of 2–3 days (Dahlstrand et al., 1995). These arrive by specific trafficking through the remote blood-CSF barrier at the ventricular choroid plexus of the brain (Damoiseaux et al., 1994) as well as through any disruption in the BB resulting from the injury itself. The oxidative and proteolytic enzymes produced by infiltrating neutrophils prepare the area for repair, however the overwhelming numbers that are drawn to the lesion can cause further damage to the surrounding tissues (Fleming et al., 2006). Macrophages and microglia are capable of producing factors that will promote axonal growth and regeneration, as well as molecules that are neurotoxic (Fleming et al., 2006).

That the profile, as well as timeframe, of the inflammatory response to SCI differs between young and adult mammals in terms of both cellular and molecular responses (Kumamaru et al., 2012) has been shown using rat models (Brown et al., 2005; Lane et al., 2007). Rodent models have also shown that microglia, the endogenous innate immune cell responders, initiate the production and secretion of cytokines into the cellular microenvironment within hours (Kumamaru et al., 2012). The profile of these cytokines, and the expression of inflammatory molecules, differs markedly between adult and infant animals (Lane et al., 2007; Kumamaru et al., 2012). The secretion of pro-inflammatory cytokines, in contrast to the anti-inflammatory, was markedly decreased in young animals (Kumamaru et al., 2012). This may contribute to the attenuation of the subsequent neutrophil infiltration that is observed in neonate mice (Kumamaru et al., 2012). High neutrophil infiltration has been found to contribute to the severity of the secondary insult (Donnelly and Popovich, 2008). Studies have found differences in the macrophage and activated microglial response in young subjects after SCI. After SCI microglia are activated as early as 24 h with a markedly less pronounced reaction in young animals compared with the adult counterparts (Vega-Avelaira et al., 2007) Different subsets of macrophages exhibit different effects within the lesion and on the progression of the recovery (Kigerl et al., 2009; David and Kroner, 2011). Classically activated (M1-like) macrophages respond to and release pro-inflammatory cytokines and also produce proteases, cytotoxic effectors and reactive oxygen species that contribute to the secondary injury (Stollg and Jander, 1999). In contrast alternatively activated (M2-like), macrophages help promote a more supportive trophic tissue microenvironment and facilitate neuronal survival and sprouting (Stollg and Jander, 1999).

Neural progenitor cells (NPC) are a population of partially differentiated stem cells that are committed to the neural line. These cells are located endogenously in niches within the CNS and have the limited self-renewing capacity and multipotency of committed stem cells (Gage, 2000). Relatively quiescent in normal CNS tissue, NPC become active and proliferate in response to trauma (Meletis et al., 2008). The majority of research regarding NPCs has been performed in rodent models. Most of these studies have found NPCs to generate a majority of cells with an astrocytic phenotype and some oligodendrocytes and OPC, similar to the behavior of transplanted cells (Barreiro-Iglesias, 2010). A proliferative burst occurring in CNS-derived progenitors as early as 24 h post-SCI has been demonstrated however the cells differentiated along glial lines with no evidence of neural differentiation (Horky et al., 2006). One potent exception to this is a study by Guo et al. (2014) that was able to induce neurogenesis using Sox11 transfected via a lentiviral vector. In Meletis et al. (2008) found that SCI induces the proliferation of ependymal cells and their migration toward the lesion site.

In recent years a high degree of interaction and “cross-talk” has been discovered between the nervous and immune systems (Ziv et al., 2006; Cusimano et al., 2012; Kokaia et al., 2012). This is highlighted by the sharing of multiple signals and pathways between the two systems (Hohlfeld et al., 2007) and has also led to the emergence of a significant role for NPC as mediators and modulators of neuroinflammation and the immune response within the CNS. NPC can exert a beneficial effect post-trauma through immunomodulation and the provision of trophic support for the surviving neurons and glia (Kokaia et al., 2012). This places greater emphasis on the synergy between these progenitors and the cells of the immune system as a therapeutic avenue, rather than the use of NPCs for cell replacement, and highlights a potential new role for NPCs after SCI. This study investigates the temporal response of innate inflammatory cells, reactive astrocytes and NPC following injury in rats of different maturities to determine where the significant differences lay and how this may translate into the difference in recovery that have been broadly observed.

## Materials and methods

### Animals

A total of 108 Sprague-Dawley rats (ARC, Perth) were used for this study. This included three age groups; infants (postnatal day 7), juveniles (5 weeks old, female) and adults (9 weeks old, female). Females were used for the adult and juvenile groups, while both the male and female pups were used for the infant groups. Within each age group rats were euthanized at four different survival times after the induction of a spinal cord injury. These were 24 h, 1, 2, and 6 weeks. There was an N of 4 for all sham groups and an N of 6–8 for all injured groups. This was approved under the University of Technology Sydney ACEC.

### Surgery and euthanasia

Experimental animals underwent either a surgically induced SCI or a laminectomy only (sham). Prior to the procedure local anesthetic and iodine were applied to the shaved thoracic incision site. The rats were administered analgesics (Buprenorphine hydrochloride -Temgesic 0.03 mg/kg), antibiotics (Cephazolin sodium 33 mg/kg) and Hartman's fluid replacement solution (Compound sodium lactate 15 ml/kg) via subcutaneous injection. An incision was made through the skin at the dorsal midline, from the mid to low thoracic region, and the subsequent layers of tissue parted by blunt dissection to expose the spinal column. This was followed by a bilateral laminectomy of the T10 vertebrae. The rats were then moved to the MASCIS impactor device (Basso et al., 1996), stabilized with clamps on the T9 and T11 vertebrae, and subjected to a weight-drop contusion injury. The height of the drop and diameter of the impactor head varied between groups to account for different sizes of spinal cords in order to produce a comparable injury severity in animals of different sizes. To induce a mild injury in the infants a weight with a diameter of 1.5 mm was dropped from 3.0 mm above the exposed cord, for juveniles a 2.0 mm head was dropped from 6.25 mm and for the adults a head measuring 2.5 mm dropped 6.25 mm.

Post-surgery the animals received fluid replacement through Hartman's Solution (1 ml/100 g S.C) as well as subcutaneous antibiotics (Cephazolin sodium 33 mg/kg s.c), and analgesics (Temgesic 0.03 mg/kg s.c). The animals were euthanized at 4 time points post-surgery, 24 h, 1, 2, or 6 weeks, using Lethobarb (pentobarbitone, 1 ml/100g, i.p). Animals were then transcardially perfused with heparinized saline followed by a 4% Paraformaldehyde solution. The spinal cords were excised and post-fixed for 24 h before storage.

### Tissue sectioning

The spinal cords were sectioned frozen using a Shandon Cryotome E cryostat. A 1 cm section of the thoracic spinal cord, approximately 5 mm on each side of the lesion center at T10. This was cut into transverse sections of 15 microns thick, every third section of which was collected. The sections were mounted in series of 10 gelatinized slides each slide giving a cross-section of the injury of approximately 5 mm (2.5 mm each side of the lesion center). This resulted in the collection of 15–20 sections through the lesion for each slide in the series. Each animal was identified only by a unique number, blinding the experimenter to the condition of the animals for the completion of all of the analyses, except for the low power imaging of lesion area when it was obvious which were the infant spinal cords due to their small size.

### Lesion size and injury markers

One slide in the series was stained using Mayer's Haematoxylin and Eosin to examine injury progression. Images were taken of each section using a PixeLINK camera attached to an Olympus BH-2 light microscope. Using the analysis program Image J the total transverse area of each of the sections on the slide, and the area of the evident tissue damage that constituted the lesion, was measured. From this the lesion area was a calculated as a percentage of the total area. The lesion consisted of the region displaying disrupted, pale necrotic tissue, picnotic nuclei, hemorrhage or a cystic cavity. This differed at each survival time point as the injury developed. Difficulties were encountered when assessing the lesion in the infant groups after 24 h survival time (1, 2, and 6 week groups) due different manifestations of the injury. In these groups there was no distinct lesion; rather the injury manifested in distorted development across the midline. This was measured as a percentage difference between the two halves, using the ventral fissure to determine the midline. The swollen axons that resulted from the trauma, were quantified as axons/100 μm^2^ (0.10 mm^2^) in the white matter of three sections on each slide. Swollen axons were counted as those individually visible under 4000× magnification, usually surrounded by a small lumen. Examples of the morphology of these are attached to **Figure 2**. This analysis was performed using the Olympus light microscope on the 40× objective at the lesion center, and approximately 2.25 mm caudal and 2.25 mm rostral to the lesion center, by an experimenter blinded to the experimental conditions of the animals.

### Immunohistochemistry

Immunohistochemical staining was undertaken for neural progenitor cells (Nestin), astrocytes (GFAP) and phagocytes (ED1/IBA1) (Frisén et al., 1995; Wei et al., 2002; Pekny, 2003). The slides were immersed in pH7.4 PBST (phosphate buffered sodium and Triton-X) for 10 min. Following PBST buffer the slides were incubated in 5% NGS (normal goat serum) as a blocking agent for 30 min. The primary antibodies were diluted in PBG (phosphate buffered goat serum) as specified in Table 1 and applied to all slides except the negative control. PBG alone was applied to this control. the primary antibodies were incubated overnight at 4°C.

**Table 1:** **Primary and secondary antibodies used for immunohistochemical staining**.

**Primary antibody**	**Secondary antibody**	**Tissue element**
Mouse anti-Nestin (Abcam, 1:200)	AlexaFluor 568 anti-Mouse (Invitrogen, 1:1000)	Neural progenitor cells (Dahlstrand et al., 1995; Xu et al., 2008)
Rabbit anti-GFAP (Dako, 1:1000)	AlexaFluor 488 anti-Rabbit (Invitrogen, 1:200)	Astrocytes (Eng, 1985)
Mouse anti-ED1 (Serotec, 1:500)	AlexaFluor 488 Anti-Mouse (Invitrogen, 1:200)	Activated macrophages and microglia (Damoiseaux et al., 1994)
Rabbit anti-IBA1 (Abcam, 1:500)	AlexaFluor 568 anti-Rabbit (Invitrogen, 1:200)	Ramified and activated microglia (O'Carroll et al., 2013; Le Blon et al., 2014)

The slides were washed in changes of PBST before incubating the secondary antibodies, diluted in PBG, for 2 h at room temperature. This was followed by further washes of PBST before counterstaining in Hoechst solution (Invitrogen, 1:5000) to stain the nuclei. Finally, all of the slides were washed in PBST and cover-slipped using Dako fluorescent mounting medium.

### Data analysis

Images were taken at 400× magnification using an Olympus DP70 camera with the black balance adjusted to control for variance between sections. This was repeated on three sections, the lesion center as well as 2.25 mm caudal and 2.25 mm rostral to the lesion center. Due the necessary thickness of the frozen spinal cord sections it was not possible to count individual cells stained for Nestin and GFAP. As an alternative the mean grayscale value (GSV) was used as a measure of the fluorescence in each section. The fluorescent intensity was used to demonstrate the expression of Nestin or GFAP. The NPC were measured using the Nestin GSV calculated for the central canal, as well as areas of 150 μm^2^ in different anatomical locations within the transverse sections. The activation and proliferation of astrocytes was measured using the GFAP GSV in areas of 150 μm^2^ in the lateral and ventral white matter and at the edge of the lesion on the three transverse sections used for the Nestin analysis. On the ED1/IBA1 double-labeled slides a single section at the lesion center was used for absolute counts of three subtypes of macrophages/microglia. These were counted based on the visible fluorescent expression of ED1^+^/IBA1^+^ (phagocytically activated microglia/macrophages), ED1^+^/IBA1^−^ (phagocytic macrophages/monocytes) (Ajami et al., 2011; Chiu et al., 2013), and ED1^−^/IBA1^+^ (ramified microglia) and also the expected morphology of activated phagocytes and resting microglia. These were not stereological counts. From these absolute counts a proportion of the total macrophage/microglial numbers was established. The data was analyzed using two-way ANOVA (using age and time as the main effects) with the Bonferonni *post-hoc* test, where the data was continuous and assumed to be of a normal distribution (GraphPad Prism software version 6).This analysis was done blinded to age and injury status as much as possible and the shams were used for direct comparison to the injured animals in each age group. All graphs depict data means and use error bars of the Standard Error of the Mean (SEM).

## Results

### Lesion size and location

**Figure 1:**
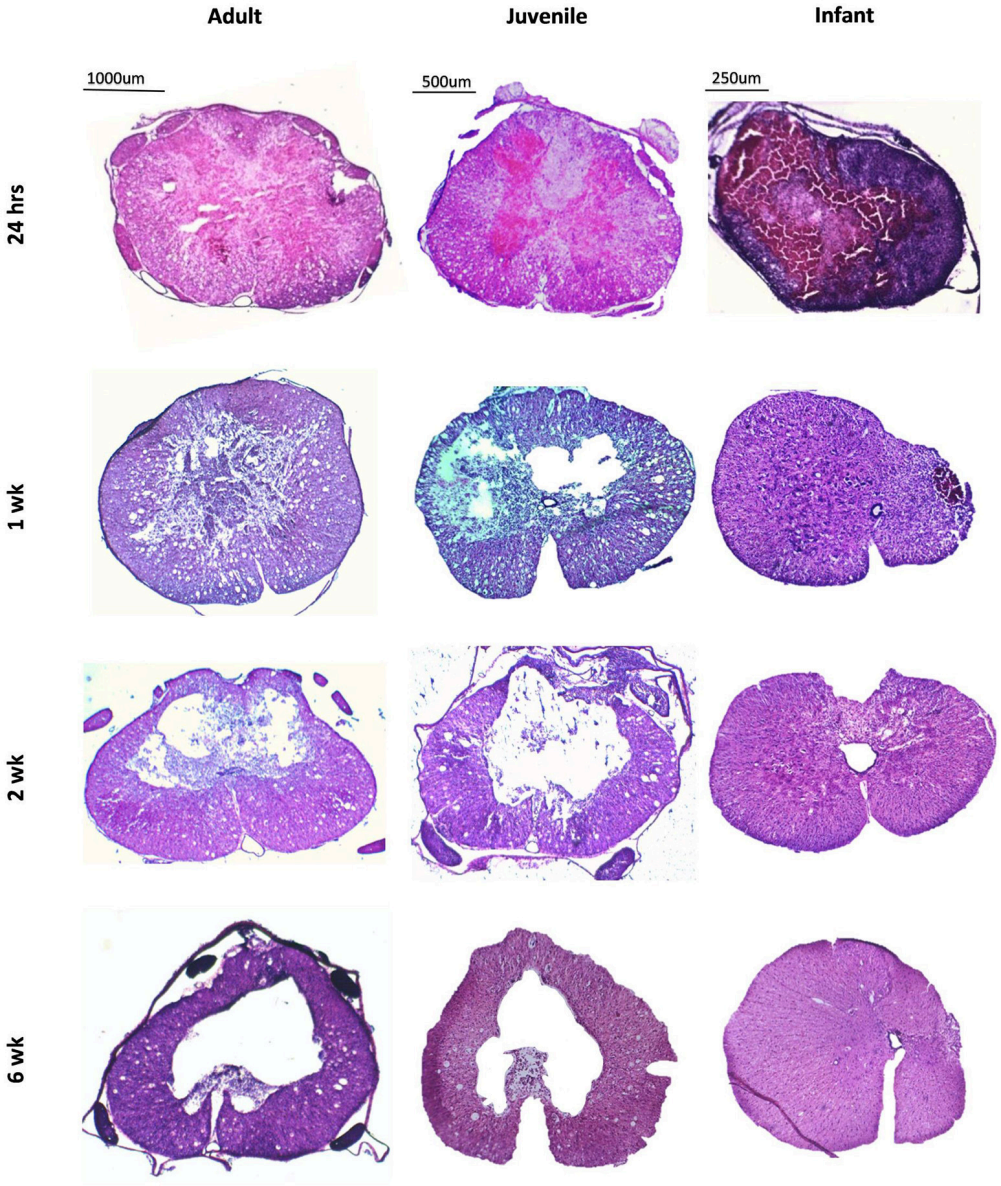
**Low power images of coronal sections of rat spinal cord following a T10 contusion injury stained with Haematoxylin and Eosin**. Images are taken from the lesion center in adult, juvenile and infant spinal cords at four time points post injury; 24 h, 1, 2, and 6 weeks. All lesions show hemorrhage and tissue disruption at 24 h post injury. The adult and juvenile rats show increasing cavitation from 1 week, whereas the infant cords exhibit marked asymmetry after 1 week.

The spinal cord lesion induced in this study present differently in infants compared to adults and juveniles (Figure 1). This is subtle at 24 h post injury. At the later time points; 1, 2, and 6 weeks, there is a stark difference in appearance of the injured cords. In the adults and juveniles there was an area of necrosis, disrupted tissue and hemorrhage that comprised the lesion at 24 h, and significant amounts of swollen axons in the intact white matter. This area became sparser at 1 week post injury and developed into a cystic cavity by 2 weeks, presenting as a large hole in the cross-section of the tissue, tapering out caudal and rostral. The infants presented with a similar lesion at 24 h post injury, with more significant hemorrhage. The infant groups, however, do not present with a cavity or disrupted lesion from 1 week onwards. Instead these animals show a disparity in the size of the left and right sides of the midline of the transverse spinal cord.

**Figure 2:**
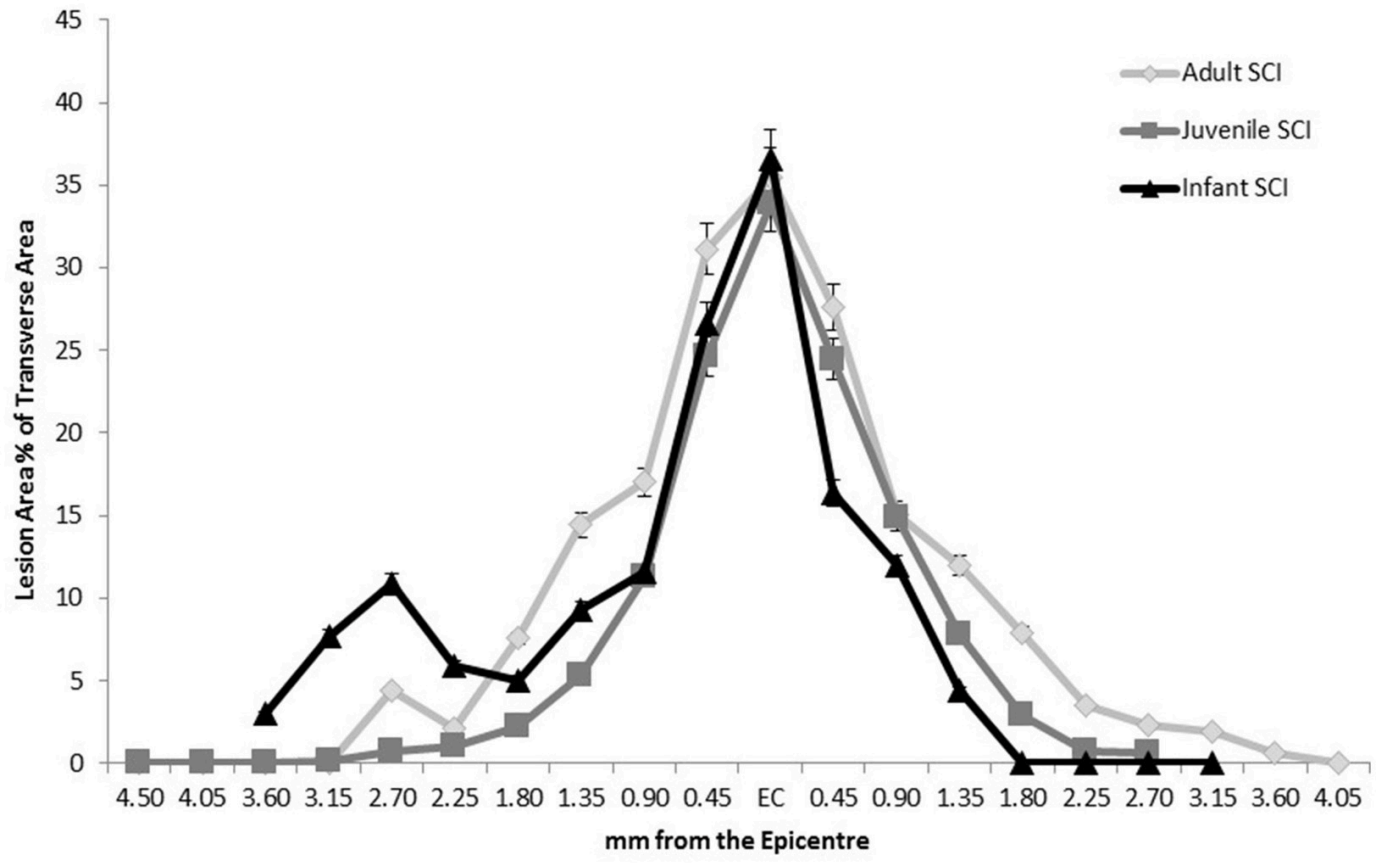
**Comparison of the extent of the lesion along the spinal cord length in all three age groups at 24 h post-injury, showing a similar pattern of injury therefore confirming a comparable injury**. Sham and control groups are not shown as they were 0 with no variance.

The measurement of the lesion as percentage of the total transverse area, or in the case of the later infant groups, the percentage difference in size between the right and left sides of the midline followed the pattern of greater disruption at lesion center tapering off distally, eventually disappearing out of the transverse sections. This happens around the 2.25 mm point generally although there is a high degree of variation between animals. There was no statistically significant difference between adult and juvenile groups at all four time points and the infants followed the same pattern, although they could not be directly compared after 24 h. This is indicative of a comparable injury model between age groups, despite the different presentations at later time points (Figure 2).

### Swollen axons

**Figure 3:**
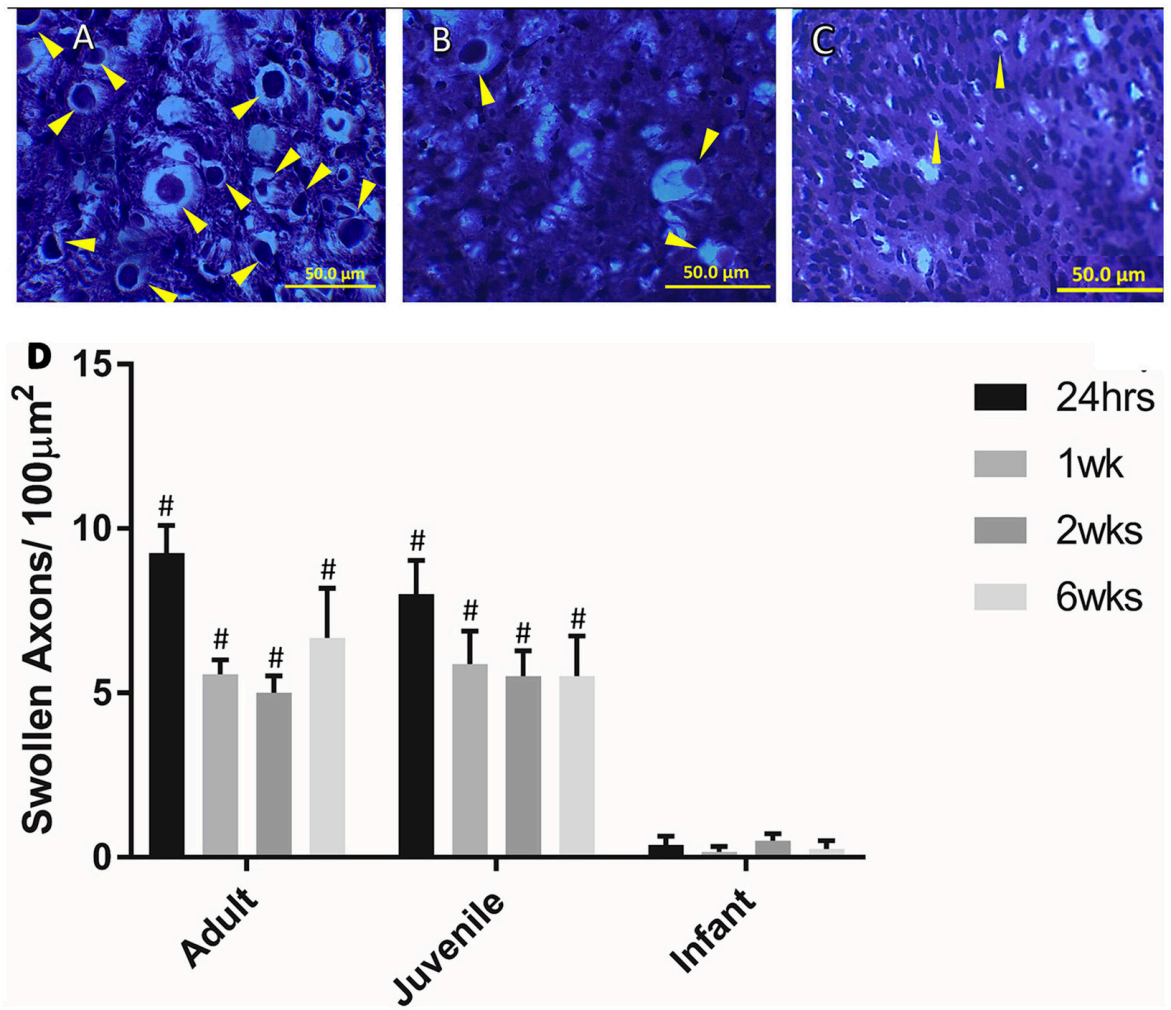
**Examples of swollen axons in (A)** Adult, **(B)** Juvenile, and **(C)** Infant white matter 1 week post injury. **(D)** Histogram of the number of swollen axons per 100 μm in the lateral and ventral white matter at the lesion center by age group, at 24 h, 1, 2, and 6 weeks post injury. # indicates a statistically significant increase (*P* < 0.001) in swollen axons in the adult and juvenile groups compared to the infants. Controls are not shown as they did not show any swollen axons. Yellow arrows indicate typical swollen axons.

Swollen axons are a common marker of trauma in the central nervous system. There were higher numbers of swollen axons at the lesion center than at the distal locations in the adult and juvenile subsets of animals, however, these differences were only significant at 24 h (Figure 3). The infants had less swollen axons/100 μm^2^ than the adults and juveniles at all four time points (*P* < 0.05). This adds another dimension to the differences in injury presentation between infants and older animals. The significant decrease in the numbers of swollen axons observed in the infants is potentially linked to the developmental state of the spinal cord.

### Innate immune response

**Figure 4:**
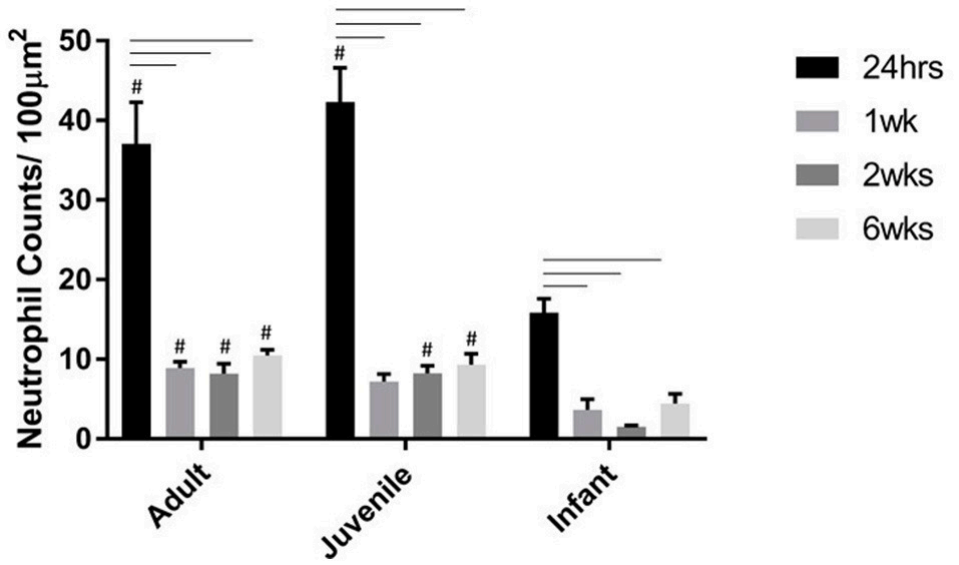
**Histogram of the number of neutrophils per 100 μm^2^ at the lesion center of the spinal cord at 24 h, 1, 2, and 6 weeks post injury**. Using two-way ANOVA there was significant difference (*P* < 0.0001) found in both main effects, age and survival time, as well as the interaction (*P* < 0.0006). There was a significant difference (*P* < 0.001) between the 24 h time-point and the later time-points. # (*P* < 0.005) indicates a significant difference between the infants and the adult and juvenile groups based on Bonferroni's *post-hoc* test.

Neutrophils are one of the first extrinsic responders to spinal cord injury and numbers were highest at 24 h post injury for all three age groups as expected. There were no neutrophils seen in any of the sham spinal cords at any time, or age groups. There were significantly higher numbers infiltrating into the injury site at 24 h compared to 1, 2, and 6 weeks post-injury (ANOVA, Bonferroni's *post-hoc P* < 0.0001). The neutrophil numbers in the adults and juveniles were substantially higher than in the infants at nearly all points (ANOVA *P* < 0.05). There were significant differences between the adult and infant groups at all four time points, as well between juvenile and infant at all time points except 1 week (Figure 4).

**Figure 5:**
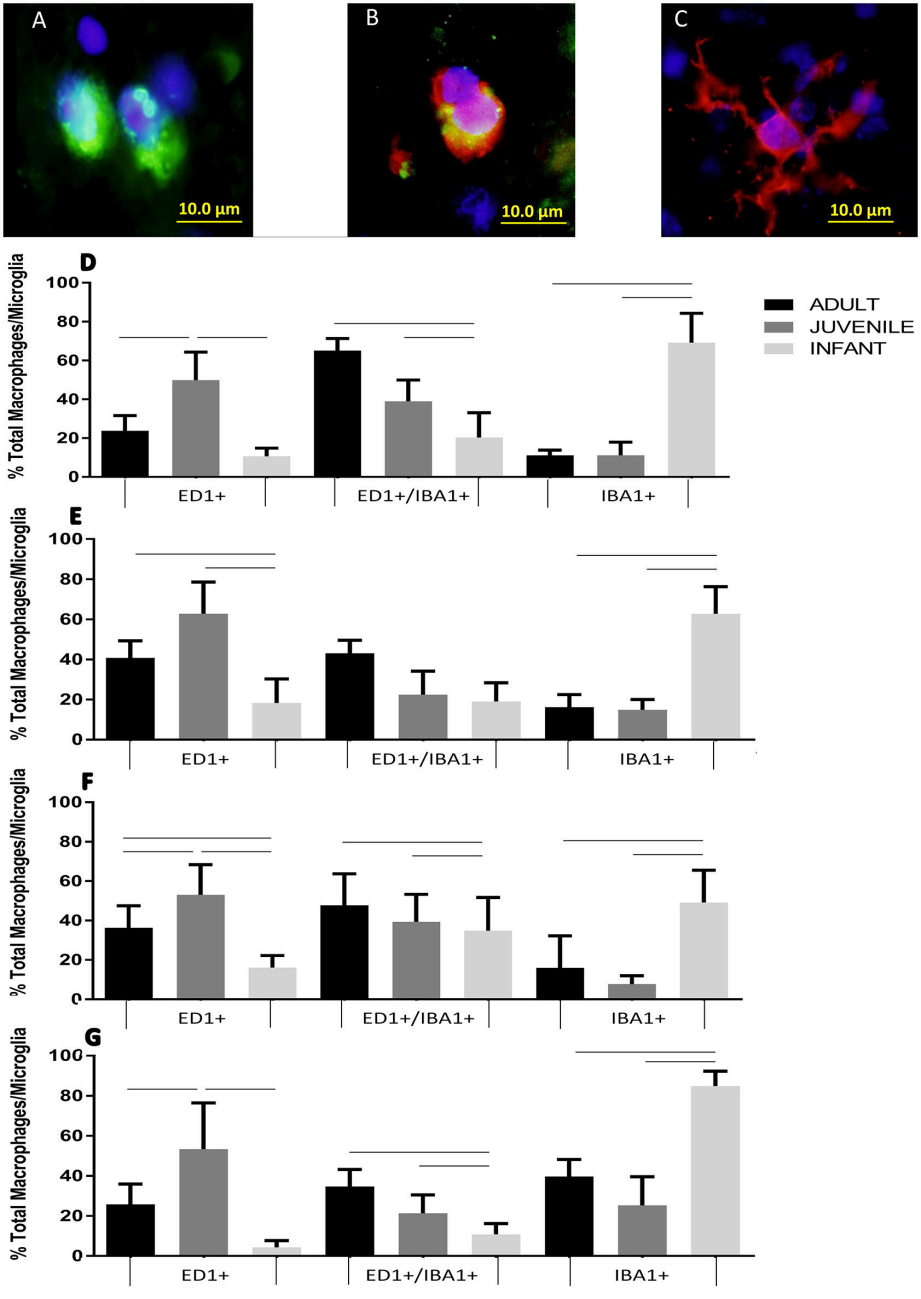
**Example fluorescent images of (A)** ED1 positive phagocytes, **(B)** EDI/IBA1 duel positive phagocytes, and **(C)** IBA1 positive ramified microglia. Histograms of the proportion of the total macrophage/microglial population at the lesion center of the spinal cord stained with ED1^+^/IBA1^−^ (ED1^+^), ED1^+^/IBA1^+^ and ED1^−^/IBA1^+^ (IBA1^+^) at **(D)** 24 h, **(E)** 1 week, **(F)**, 2 weeks and **(G)** 6 weeks post-injury. Significant differences (*P* < 0.05) between groups are shown based on Bonferroni's *post-hoc* test.

Extrinsic macrophages and intrinsic microglia are also inflammatory responders belonging to the innate immune system. In the sham groups cells had a predominant ED1^−^/IBA1^+^ profile, representing the normal microglial population, at all time points, and at all ages, and hence are not shown in Figure 5. For the injury groups there was an opposing pattern of ED1/IBA1 staining in the infants compared to the adults and juveniles at all four survival times. The adult and juvenile groups showed increases in the proportions of ED1^+^/IBA1^−^ (phagocytic macrophages/monocytes) and ED1^+^/IBA1^+^ (activated microglia) and decreases in the proportion of ED1^−^/IBA1^+^ (ramified microglia) compared to the shams, which was largely found to be statistically significant. The infant, on the other hand, presented with only small increases in the proportion of the two ED1^+^ phagocytic subsets and a small decrease in the proportion of ED1^−^/IBA1^+^ resting cells from the sham levels, very little significance was found in these changes. There was statistical significance (*P* < 0.05) found in the differences between the age groups especially with the proportions of ED1^+^/IBA1^−^ and ED1^−^/IBA1^+^ cells, at all four time points (Figure 5).

### Reactive astrogliosis

**Figure 6:**
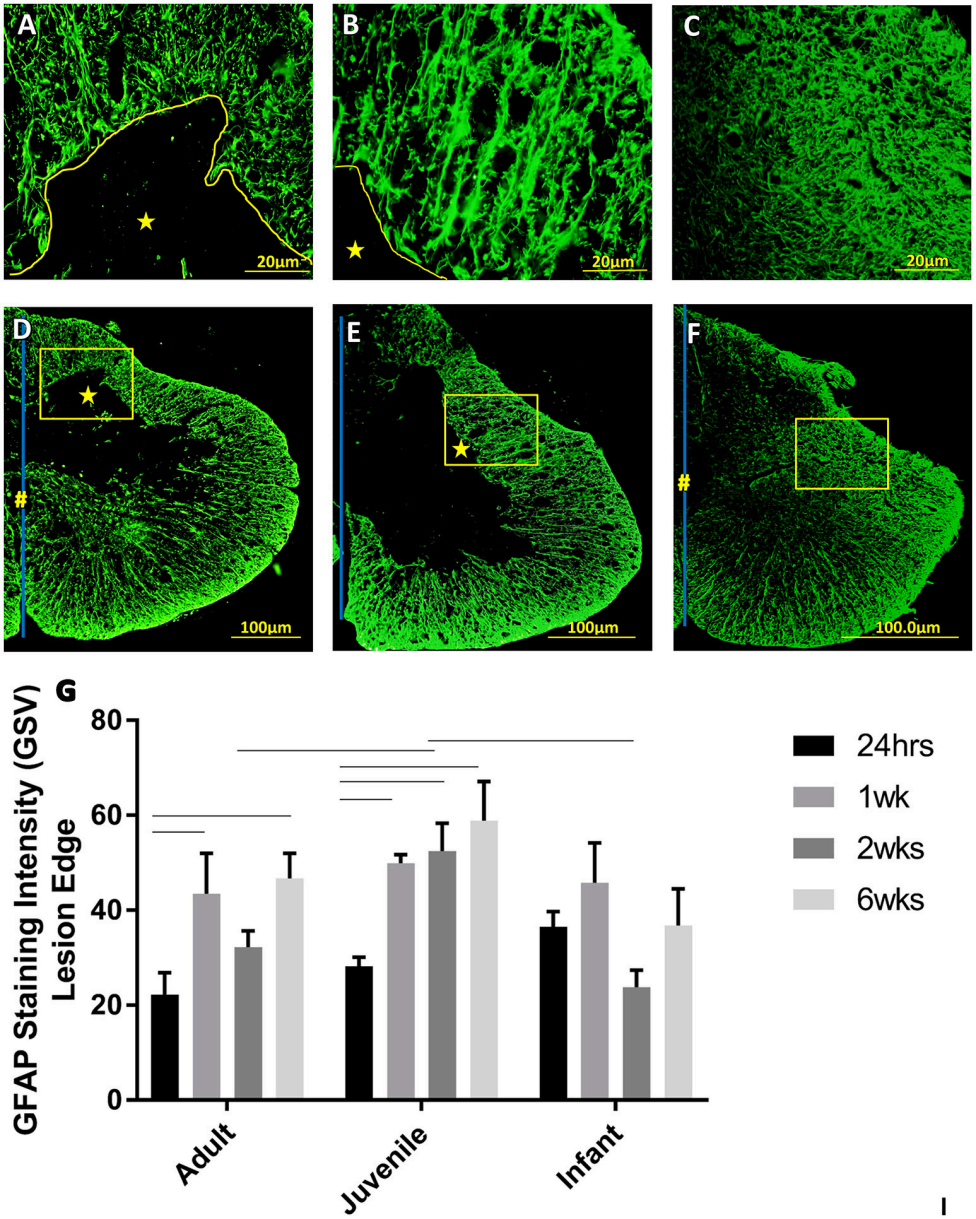
**Representative high power fluorescent images of astrocytic processes at the lesion edge in (A)** Adults, **(B)** Juveniles, and **(C)** Infants. These represent the areas denoted by the squares in low power images of one side of the transverse spinal cord shown in **(D)** Adults, **(E)** Juveniles, and **(F)** Infants 1 week post injury. This demonstrates astrogliosis around the lesion and astrocytes in the spared white and gray matter stained with GFAP (green). The lesion is denoted by ^*^, the central canal is indicated by # where it is intact and the midline by the blue line. **(G)** Histograms of the intensity of glial fibrillary acidic protein (GFAP) staining (mean grayscale value) at the edge of the lesion at 24 h, 1, 2, and 6 weeks post-injury. Using two-way ANOVA there was significant difference found in both main effects, age (*P* < 0.005) and survival time (*P* < 0.0002) as well as the interaction (*P* < 0.05). Significant differences (*P* < 0.05) between groups are shown based on Bonferroni's *post-hoc* test.

Astrocytes are prominent glial cells in the spinal cord that respond over a longer time course after SCI to eventually form a glial scar around the lesion site. At 24 h after the induced SCI there is a small increase in the GFAP around the lesion edge, however this was not statistically significant (Figure 6). The glial scar is a chronic injury resolution mechanism and, as indicated by this data, is not yet present at this early a time point. One week after SCI the GFAP is still increasing at the lesion edge as the glial scar begins to form. This is fairly consistent between the age groups with no significant differences found in the GFAP intensity around the lesion edge (Figure 6). Two weeks post injury the GFAP around the lesion edge is still increased in the adult and juvenile groups but the infants have returned to sham levels. The juvenile rats are the only group to show significant increases from the sham level at all three locations on the cord, however there is still a visible increase in the adults as well (Figure 6). By 6 weeks post injury the adult and juvenile rats have significantly increased GFAP intensity around the lesion edge at all three locations on the spinal cord tested, compared to their respective shams, consistent with the formation of the glial scar. The injured infants, however, are consistent with their sham counterparts (Figure 6).

The white matter is where the GFAP staining is most prominent in a normal cord. In the white matter at 24 h post injury there is a visible increase in the GFAP, however this is not statistically significant. The only statistically significant difference is between the GFAP in the infant and adult white matter, with the infants having significantly higher GFAP intensity. By 2 weeks into the injury progression the GFAP intensity has returned to sham levels in all three age groups, at all spinal levels.

### Endogenous neural progenitor cells

**Figure 7:**
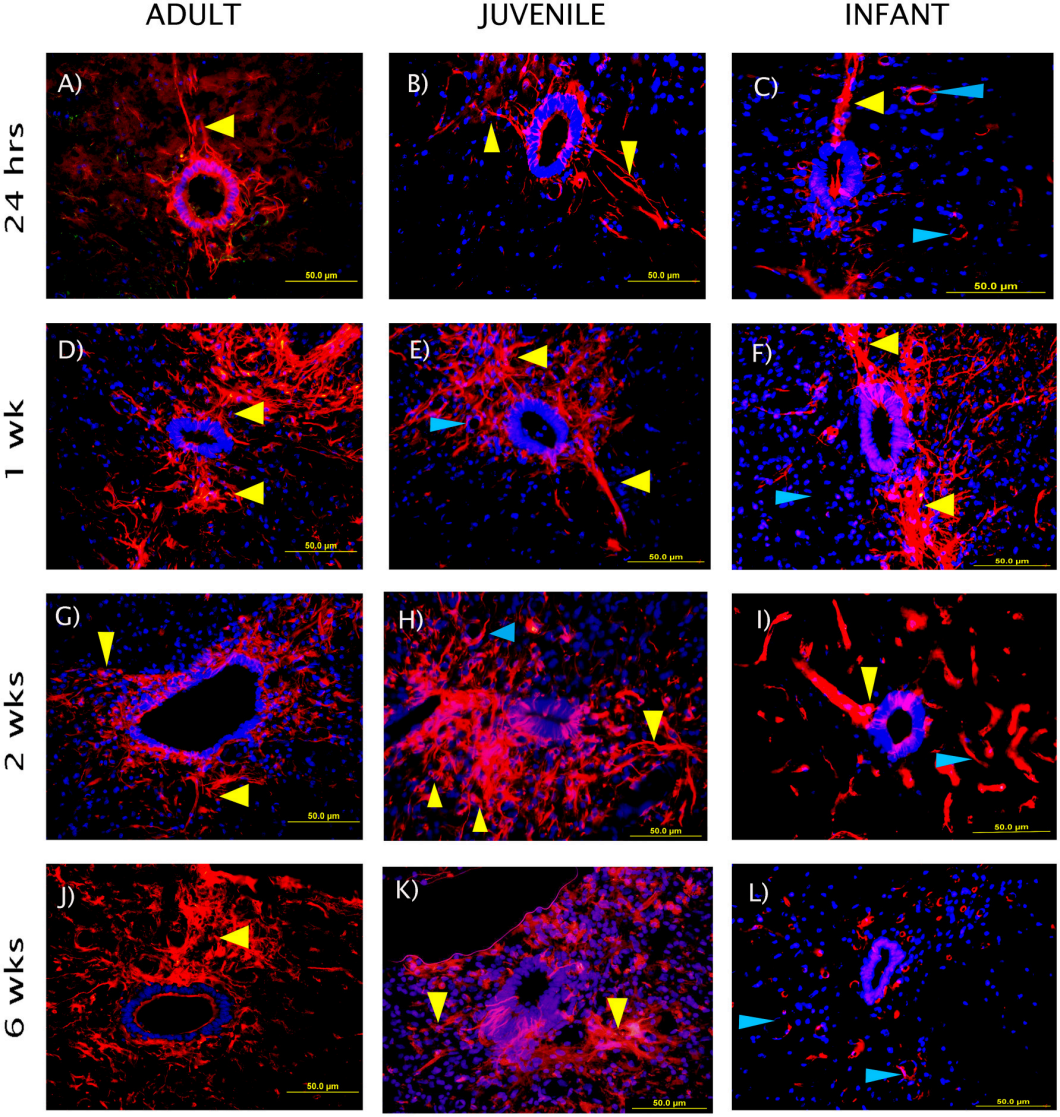
**Representative fluorescent microscopy images of the central canal taken at 400×, 2.25 mm distal to the lesion center, stained with nestin as a marker for endogenous neural progenitor cells (red)**. The nuclei are shown in blue. The ependymal layer of the central canal and long processes extending into the parenchyma can be seen (yellow arrows), some blood vessels are also visible as strongly nestin positive (blue arrows). The staining intensity in the infants is visibly lower than the adults and juveniles at the 2 and 6 week time points. **(A–C)** show the initial reaction at 24 h post-injury, **(D–F)** at 1 week, **(G–I)** at 2 weeks, and **(J–L)** depict the fading reaction at 6 weeks post-injury.

**Figure 8:**
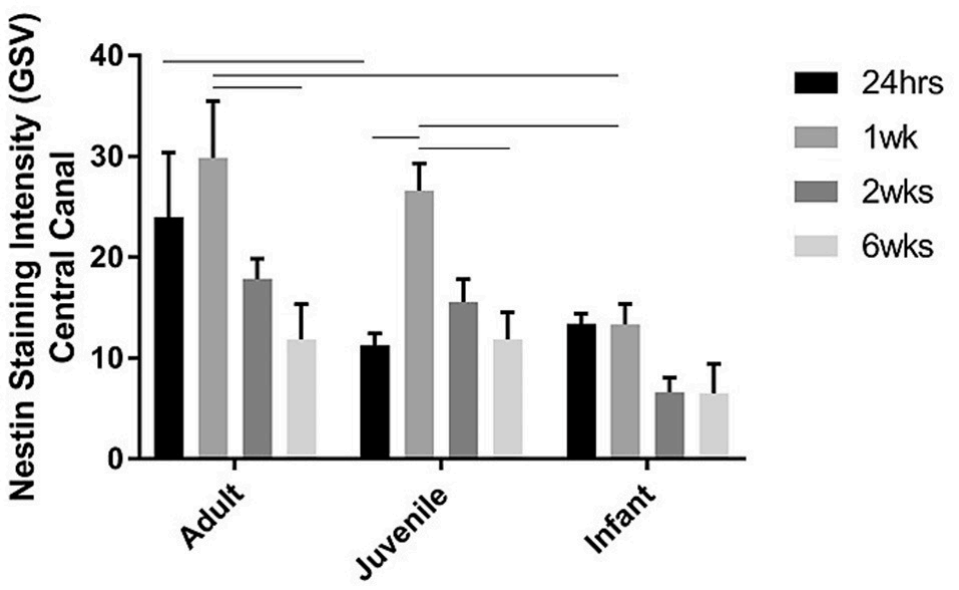
**Histogram of the intensity of nestin staining (mean grayscale value) indicating endogenous neural progenitor cells around the ependymal layer of the central canal across the lesion, at 24 h, 1, 2, and 6 weeks post-injury**. Using two-way ANOVA there was a significant difference (*P* < 0.005) in both main effects, age and survival time. Significant differences (*P* < 0.005) are shown between groups based on Bonferroni's *post-hoc* test.

The NPCs in the ependymal layer of the central canal (Figure 7) are activated at 24 h post SCI in all three age groups. This can be observed most clearly in the first row of fluorescence images in Figure 7, which shows immunohistochemically stained cells around the ependymal layer of the central canal with long basal processes extending into the parenchyma, typical morphology for activated NPCs. At 24 h post injury there was a high degree of variance between animals that resulted in less statistical significant differences between groups, however each age group showed a significant increase in nestin staining from the control levels around the central canal throughout the lesion (Figure 8). One week post injury the nestin intensity remains increased from the controls and also from the staining observed at 24 h; this represents a peak in nestin intensity around the central canal. There is a visible trend of higher nestin intensity in the adults and juveniles compared to their infant counterparts (Figures 7, 8). By 2 weeks post injury the nestin levels around the ependymal layer have begun to decrease in all three age groups; this is most significant in the infants which are almost back to sham levels (Figures 7, 8). In the chronic conditions represented by the 6 week survival groups all three ages have decreased substantially from their peak at week post injury (Figures 7, 8).

**Figure 9:**
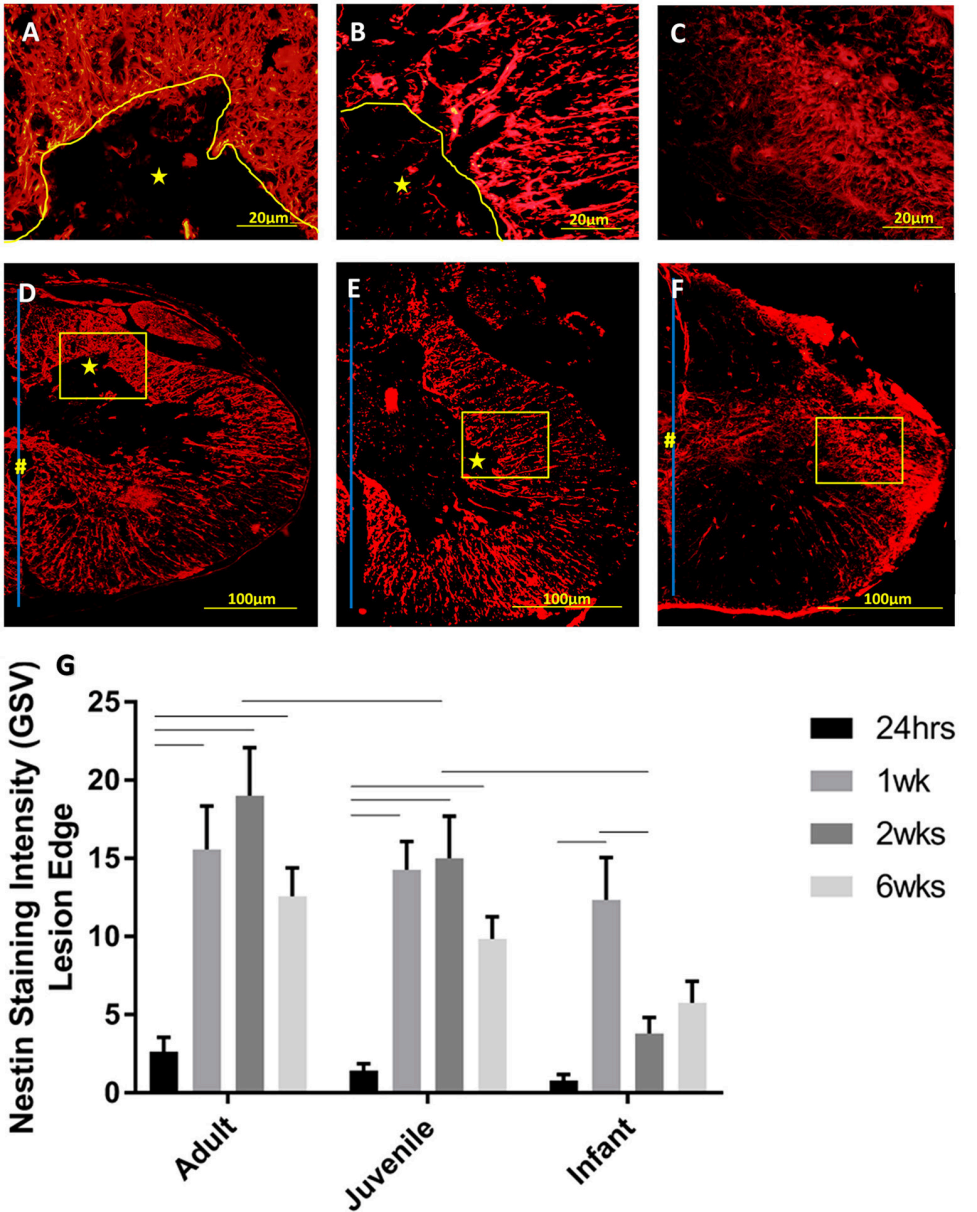
**Representative high power fluorescent images of nestin staining (red) at the lesion edge in (A)** Adults, **(B)** Juveniles, and **(C)** Infants. These represent the areas denoted by the squares in low power images of one side of the transverse spinal cord shown in **(D)** Adults, **(E)** Juveniles, and **(F)** Infants 1 week post injury. The lesion is denoted by ^*^, the central canal is indicated by # where it is intact, and the midline by the blue line. **(G)** Histogram of the intensity of nestin staining (mean grayscale value) indicating endogenous neural progenitor cells and processes around the edge of the lesion or cavity at 24 h, 1, 2, and 6 weeks post-injury. Using two-way ANOVA there was a significant difference (*P* < 0.0001) in both main effects, age and survival time. Significant differences (*P* < 0.005) are shown between groups based on Bonferroni's *post-hoc* test.

**Figure 10:**
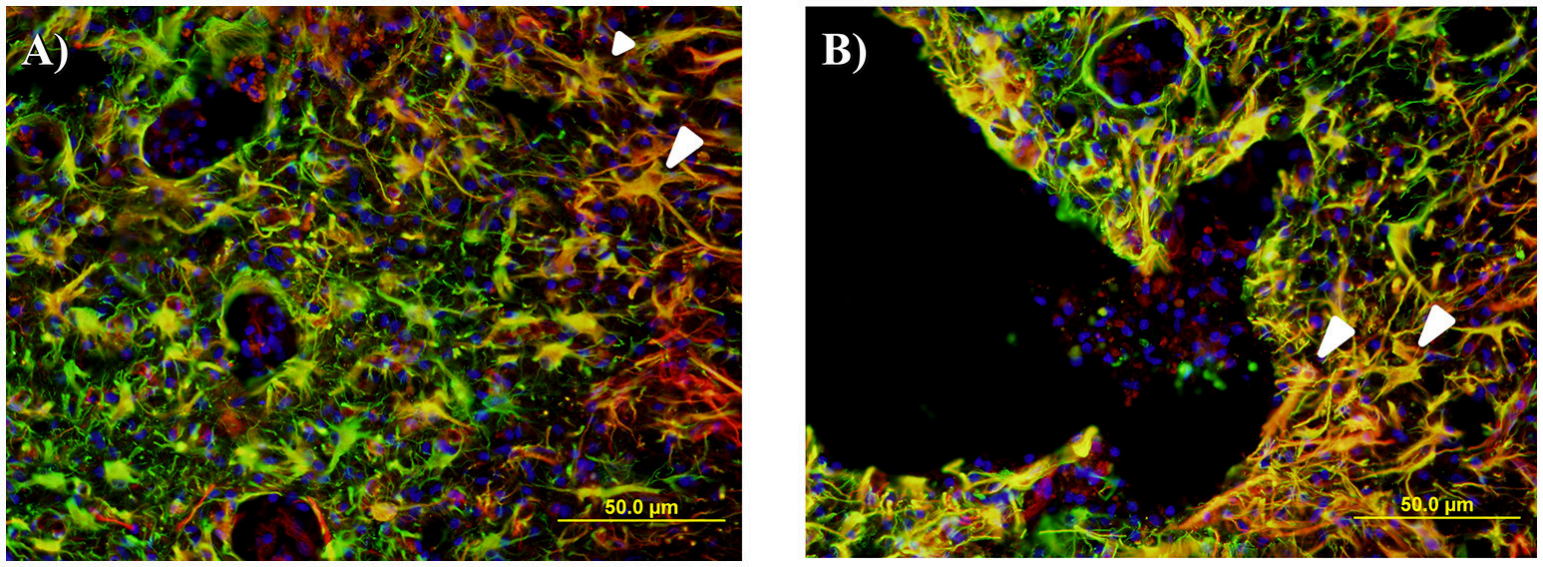
**Representative fluorescent microscopy images of gray matter (A)** and lesion edge **(B)** taken at 400×, 2.25 mm distal to the center of the injury, stained with nestin as a marker for endogenous neural progenitor cells (red) and glial fibrillary acidic protein (GFAP) for astrocytes (green). This shows a number of double labeled cells and processes in an orange color, many of which have an astrocytic appearance (white arrows).

Activated NPCs proliferate and migrate toward the injury site post-SCI (Mothe and Tator, 2005). This can be seen 1, 2, and 6 weeks post injury as the nestin staining shows significant increases around the lesion edge (Figure 9). 24 h post-injury there was no increase in the levels of nestin visible at the lesion edge (Figure 9); this may indicate that the activated cells and their processes have not yet migrated to the lesion site. Significant nestin fluorescence at the lesion edge is visible at 1 week in all three age groups and peaks at 2 weeks post injury in both the adult and juvenile rats. The nestin intensity around the lesion was highest at 1 week post injury in the infants and drops off substantially by 2 and 6 weeks. Due to the variance between animals there was little statistical significance between the age groups however the infants have visibly decreased nestin intensity, especially at the two later time points (Figure 9). Of the nestin positive cells around the edge of the lesion there was a large subset that stained with GFAP as well. These cells were not able to be individually quantified due to the thickness of the sections however many displayed an astrocytic phenotype (Figure 10).

## Discussion

### Injury progression is significantly different in infant rats

At 24 h all three age groups exhibited similar injury severity histologically, but appeared vastly different at the three later time points (1, 2, and 6 weeks). Both the juvenile and adult groups then progressed to a cystic cavity at later time points. In contrast to this, the infant groups exhibited asymmetrical tissue sparing at later time-points, but no cystic lesions. There are a few potential explanations for this phenomena; based on the increased plasticity of the infant cords as well as the developmental state of the cord at post-natal day 7. It is well established that the young and still developing spinal cord has greater neural plasticity (Bregman et al., 1993), this may allow for greater cellular survival and neural re-wiring compensating for the injury. Swollen axons are a widely used marker of CNS injury. The number of visible swollen axons was consistently higher in the adult and juvenile animals, compared to the infants. The significantly lower numbers of swollen axons observed in the infants is potentially linked to the developmental state of the spinal cord. By P7 the majority of axonal tracts should be past the T10 level of the spinal cord according to previous retrograde and anterograde tracing studies (Joosten et al., 1987; Gianino et al., 1999) as well as prior experience in this laboratory. The developmental plasticity of these axons may account for the stark differences in the injury presentation in the infant groups post 24 h in the current study. Another potential contributing factor for physical differences may be due to differences in biomechanical stresses (Clarke and Bilston, 2008; Clarke et al., 2008, 2009). It is possible that there is mechanical deformation in the younger cords in response to the dorsal impact that is quite different to that shown by adults. For example injury is greater after a dislocation injury in infant compared to adult rats, even though the young animals exhibit better recovery (Lau et al., 2013).

The measurement of the lesion as percentage of the total transverse area followed a strong pattern of greater disruption at the lesion center tapering off distally. In the case of the later infant groups the percentage difference in size between the right and left sides of the midline was measured. There was no statistically significant difference between adult and juvenile animals and the infants followed the same pattern. It is difficult to create spinal cord injuries in infants that exactly match their adult counterparts for a number of reasons including the obvious differences in animal size and weight, and also in the maturity of the CNS (Clarke and Bilston, 2008; Clarke et al., 2009; Yuan et al., 2013). The difficulties in creating a comparable infant model stem from reasons as simple as the size of the infant animal, to more complex and poorly understood factors such as the different biomechanical properties of the infant cord and how the state of development of the CNS and inflammatory response effect the injury. In this study we have attempted to create a model of mild contusion SCI across three different ages of rats. This was done by changing the size of the 10-gram impactor head for each of the ages (2.5 mm for adults, 2.0 mm for juveniles and 1.5 mm for infants) to match the size of the cord, and changing the height of the impact drop in the infants (3.0 mm drop compared to 6.25 mm). The final impact parameters used were based on a series of pilot studies aimed at matching the anatomical and histological injury severity, including a similar proportion (~70–80%) of impactor head diameter to spinal cord width for all ages (Unpublished Data). Unfortunately, the size of the neonatal rats precluded the completion of the circuit on the MASCIS to record the force, velocity or compression rate of the impact in order to compare to the adults, so the impacts themselves could not be quantitatively compared. From the similarities in lesion size and appearance at 24 h in our study (Figure 2) we have concluded that the surgically induced SCI was relatively consistent between the three age groups in terms of severity, and therefore subsequent findings can be compared between adults, juveniles and infants. This is important for the interpretation of all other data and allows for a valid comparison between the three age groups.

### The innate immune response is decreased in magnitude in infants

The long-established privileged status of the CNS has been called into question in recent years with the developing of a deeper understanding of the unique immune responses that occur in the brain and spinal cord under different pathological conditions (Ransohoff and Brown, 2012). In the early stages of development the semi-privileged status of the CNS conveys an advantage however, in pathological conditions, this differing immune response can hinder normal recovery (Schwartz et al., 1999). In the case of traumatic SCI, inflammation has been shown to have both neuroprotective and neurotoxic actions (Schwartz et al., 1999; Ekdahl et al., 2003; Popovich and Jones, 2003; Lucas et al., 2006; Hohlfeld et al., 2007; Kigerl et al., 2009; Das et al., 2012). Which of these prevails in the injured tissue microenvironment is influenced by a myriad of factors, both intrinsic to the cell types present and extrinsic within the shifting tissue microenvironment. This duality is the root of the complexity underlying the inflammatory response to traumatic injury and also in targeting elements of this therapeutically (Popovich and Jones, 2003; Hohlfeld et al., 2007). It has been established that the immature brain has a distinctive inflammatory response, compared to the adult brain, and also different vasculature (Potts et al., 2006). The inflammatory response has been observed to differ in developing spinal cords as well (Kumamaru et al., 2012). These differences are highlighted in the current study through the examination of the neutrophil, macrophage and microglial response over a 6 week time course post traumatic SCI.

In a typical response to CNS injury the first cells to arrive, within hours of the insult are neutrophils (Pineau et al., 2010; Gensel and Zhang, 2015). In terms of the inflammatory response to SCI, neutrophils play an important role and the magnitude of neutrophil infiltration may depend on the severity of the trauma (Chopp et al., 2000; Pineau et al., 2010). Neutrophils are capable of producing reactive oxygen species and other neurotoxic factors within the lesion microenvironment that can further contribute to lipid peroxidation and lesion spread in the local area (Burke et al., 2001). However, they are also the first responding cells that begin the inflammatory process in earnest and secrete chemo-attractants and activators for other important cells types (Bartholdi and Schwab, 1997; Taoka et al., 1997; Trivedi et al., 2006). These cells are expected to peak within 2 days post injury in rats (Hausmann et al., 1999; Zhang and Gensel, 2014) and persisting for weeks (Hausmann et al., 1999). In humans this happens within hours (Lucas et al., 2006). This pattern was sustained by the neutrophil quantitation in this project. There were significantly higher numbers infiltrating into the injury site at 24 h compared to the later time points (Burke et al., 2001). The potential of neutrophils for both beneficial and detrimental effects on the development of the secondary injury is dependent on the magnitude and length of the response. The oxidative and proteolytic enzymes produced by infiltrating neutrophils prepare the area for repair, however the overwhelming numbers that are drawn to the lesion can cause further damage to the surrounding tissues (Fleming et al., 2006). In the current study there were significant differences between the adult and infant groups at all time points, as well between juvenile and infant at all time points except 1 week. The infants were universally lower than the adult and juvenile groups, even when this was not statistically significant, as has been previously described for neonatal mice (Kumamaru et al., 2012). This could be of significant benefit for the infant cohort as there are still neutrophils present to play their essential role however the numbers are not overwhelming enough to have a significant detrimental effect.

Extrinsic macrophages and intrinsic microglia are also inflammatory responders belonging to the innate immune system. Microglia are a unique myloid cell population that are the innate phagocytes of the CNS, quite apart from analogous cells in other tissues (Schwartz et al., 1999; Ransohoff and Brown, 2012). These cells likely evolved as a compensatory mechanism for the unique immune status that the CNS exhibits (Schwartz et al., 1999). Extrinsic monocytes and macrophages only have access to the CNS in cases of pathology, trauma and disruption to the blood-brain barrier or blood- spinal cord barrier. Immediately following SCI an intensive inflammatory response is initiated local to the lesion, involving the activation of microglia and additional populations of macrophages from the bloodstream (Carlson et al., 1998). The action and distribution of endogenous microglia and infiltrating monocyte-derived macrophages has been shown to differ after SCI with their functions overlapping but not entirely replacing one another (Potts et al., 2006; Pineau et al., 2010; Gensel and Zhang, 2015). Both of these subsets play essential roles in the progression of injury; however, the spontaneous levels of anti-inflammatory M2-like activated macrophages infiltrating after injury may not be sufficient for efficient tissue repair (Potts et al., 2006). It must be noted that the majority of this work has been conducted in adult models with very few comparing the mature and developing (Vega-Avelaira et al., 2007; Kumamaru et al., 2012; Yuan et al., 2013).

These cells showed an opposing pattern of ED1/IBA1 staining in the infants compared to the adults and juveniles at all survival times. The adult and juvenile groups showed increases in the proportions of phagocytic macrophages/monocytes and activated microglia, and decreases in the proportion of ramified microglia compared to the shams. The infant, on the other hand, presented with only small increases in the proportion of the two active subsets and a small decrease in the proportion of resting cells from the sham levels. The different subsets of macrophage and microglia potentially have a great impact on the development of the lesion through their phagocytic role as well as the cytokines that they are secreting. Previous studies have found that profile of cytokines, and the expression of inflammatory molecules, differs markedly between adult and infant animals (Lane et al., 2007; Kumamaru et al., 2012). The secretion of pro-inflammatory cytokines, in contrast to the anti-inflammatory, was markedly decreased in young animals (Kumamaru et al., 2012). These differences between activated pro-inflammatory and anti-inflammatory phenotypes, between ages of animals, requires further exploration.

There were statistically significant differences between the age groups, especially with the proportions of ED1^+^/IBA1^−^ and ED1^−^/IBA1^+^ cells, at all four time points. This is potentially a very significant factor in the differences in the recovery between age groups; and the different injury progression observed in the H&E slides between infants and adults. The higher proportion and numbers of classically activated macrophages and microglia in the adults is likely contributing to a more robust pro-inflammatory lesion microenvironment and the detrimental propagation of the secondary injury. This pro-inflammatory cascade is initially essential in SCI to clear debris and begin the process of inflammation by the activation of other subsets of cells, and finally repair. However, if this process is sustained for too long without progressing to the tissue repair stage dominated by alternatively activated inflammatory cells, as it does in adult SCI, it becomes a detriment to tissue repair and promotes poor injury resolution. The presence of these phagocytically activated cells at lower levels in the infants may be a sign of a less robust pro-inflammatory response, with cells present in high enough levels to be beneficial but not so high as to be detrimental. While the general profile of this response is the same in the adults and juveniles there are potentially differences in the cellular profile. This can be seen in the higher numbers of solely ED1 positive cells in the juveniles while the adults displayed higher numbers of ED1/IBA1 duel positive cells. The timing of the expression of these two cell sub-types and the lack of significant differences between all of the age groups in the ED1/IBA1 subset at 1 week post injury may suggest the majority of the duel positive cells are activated microglia, while the ED1 positive subset are infiltrating macrophages.

The cellular inflammatory results of the current study strongly suggest that the differences in the innate inflammatory response cannot be overlooked when searching for the mechanisms behind the observed trend of a better outcome in infant animals and younger patients. It has been suggested that the manipulation of the inflammatory cascade toward the alternatively activated phenotype would be beneficial, and that blood-derived macrophages play a vital role in the resolution of SCI through their M2-like capabilities (Klusman and Schwab, 1997; Carlson et al., 1998; Hausmann, 2003). The elucidation of the activation phenotype of these inflammatory cells is essential to understand the contribution of each cell type to tissue repair at differing ages of animals and times post injury.

### Reactive astrogliosis follows a very similar pattern regardless of age, and the differing injury progression seen in the infant rats

Reactive astrogliosis is a well-known, widely studied process that occurs after insult to the CNS referring to the molecular and morphological changes that astrocytes undergo, including the formation of a glial scar (Sofroniew, 2009). After injury reactive astrocytes wall off the damaged areas and seal the lesion to protect the intact tissue from further damage (Fitch and Silver, 2008); however, they are not simply inert structural elements, they also secrete a variety of molecules into the lesion microenvironment. Like many elements of the post-injury tissue microenvironment the response of reactive astrocytes has both beneficial and detrimental aspects (Fawcett and Asher, 1999; Faulkner et al., 2004; Profyris et al., 2004; Silver and Miller, 2004; Sofroniew, 2009). *In vitro* studies have previously shown that astrocytes produce effector molecules, both pro- and anti-inflammatory, that can both help and hinder functional recovery (Silver and Miller, 2004). The debate continues as to whether the process of reactive astrogliosis, and the eventual formation of a glial scar, are beneficial or detrimental after SCI (Fawcett and Asher, 1999; Profyris et al., 2004; Silver and Miller, 2004; Fitch and Silver, 2008; Sofroniew, 2009) with much of the literature expounding on both neuroprotective and inhibitory elements of the glial response (Kwon et al., 2004; Sofroniew, 2009).

There are regional differences in GFAP intensity within the transverse sections that mirror the anatomy of the spinal cord, with higher densities of astrocytic projections in the white matter than the gray matter (Yang et al., 1993; Baldwin et al., 1998). Following from this, the most prominent changes in GFAP staining intensity were noted in the white matter as well as around the lesion edge as the glial scar forms. As the infant cords are still in a developmental state it was expected that they would have a higher astrocytic density to begin with (Yang et al., 1993; Vega-Avelaira et al., 2007). This was borne out by the results of the current study showing a higher immunoreactivity in the infant groups, both injured and shams, when compared with their respective adult and juvenile counterparts.

The glial scar had not begun to form around the lesion at 24 h; although there was a slight observed increase in astrocytic density indicated by a small increase in GFAP immunoreactivity. This is to be expected as astrocytes typically react to CNS injury over the course of days to weeks, so at this acute time point in the development of SCI the astrocytic response will be only in its preliminary stages (Profyris et al., 2004; Silver and Miller, 2004; Fitch and Silver, 2008). At 1 week after injury the adult and juvenile animals showed significant increases from the control GFAP at the lesion edge, representing the beginning of the glial scar structure and a peak in fluorescent intensity as the reactive astrocytes are proliferating and in a highly active state. The infants demonstrated some increase however it was to a lesser degree and not statistically significant. These results highlight another difference in the response to SCI between infant and adult animals, likely intrinsically connected to the stark differences in the histological progression of the injury. The infant animals did not exhibit a lesion or cystic cavity after 24 h post-injury and so there is no distinct injury site to be walled off. That said, astrocytes do still play a role in the post-injury tissue microenvironment as there was an increase in GFAP where the lesion edge would have been, even though this was not considered to be significant.

Lane et al. (2007) found that the accumulation of GFAP-positive astrocytes at the lesion site occurred much earlier in opossums injured at post-natal day 14 (P14) than those injured at P7. This led to the idea that the delayed timeframe of activation and migration of reactive astrocytes in young subjects may also contribute to the faster and fuller functional recovery of young animals, alleviating some of the negative effects of the glial scar. The results of our experiments agree with this contention, in that the levels of GFAP were starkly and significantly increased at 24 h, compared with the adult and juvenile animals. However, our findings differ from Lanes in that GFAP at the lesion edge remained unchanged. This could be a result of the different mode of injury (contusion rather than complete transection) and the use of transverse rather than longitudinal sections.

### Endogenous neural progenitor cells are viable therapeutic targets

Several studies have reported a proliferation and differentiation in neural progenitors as early as 24 h post-SCI with cell differentiation into glial cells and oligodendrocytes, but no evidence of neurogenesis (Horner et al., 2000; Cao et al., 2001; Tzeng, 2002; Mothe and Tator, 2005; Horky et al., 2006; Hamilton et al., 2009; Marichal et al., 2009; Barnabé-Heider et al., 2010; Mothe et al., 2011). The NPCs in the current study are activated at 24 h post injury in adult, juvenile and infant rats. This is demonstrated by the significant increase in the nestin level in the ependymal layer of the central canal, as previously observed in adult rats (Mao et al., 2016). Nestin positive cells were shown by Horky et al. to persist in the parenchyma for up to 9 weeks post injury (Horky et al., 2006) which is supported by the persistent nestin staining in the central canal and lesion edge that was seen up to 6 weeks post injury in the current study, in the adult and juvenile. The persistence of the nestin staining in the infants was substantially lower, especially in the later stages of the injury progression, as the central canal levels dropped back toward normal by 2 weeks post injury. Zai and Wrathall (2005) postulated that NPC restore cell density but not necessarily functionality post-SCI, and are stimulated to proliferate in the first week post injury developing into mature phenotypes.

Activated NPCs proliferate and migrate toward the injury site post-SCI where they are thought to contribute to the glial scar (Mothe and Tator, 2005; Meletis et al., 2008). The visible nestin increases around the edge of the lesion at 1, 2, and 6 weeks post injury may be a manifestation of this, however we cannot say this for certain as reactive astrocytes can also express nestin when actively proliferating (Faulkner et al., 2004; Barnabé-Heider et al., 2010; Hu et al., 2010). This nestin increase was significant in all three ages at 1 week post injury and peaks at 2 weeks post injury in both the adult and juvenile rats. This differs from the infant rats that peaked at 1 week but drop off substantially by 2 and 6 weeks. In 2008 a study by Meletis et al. found that ependymal cells act as neural stem cells (NSC) *in vitro*. This study also confirmed that SCI induces the proliferation of ependymal cells and their migration toward the lesion site where they will also contribute to the formation of the glial scar *in vivo* (Meletis et al., 2008). Mothe et al. (2011) also support the model that ependymal cells proliferate and migrate toward the lesion in response to SCI, differentiating mostly into astrocyte phenotypes that will assist in reactive astrogliosis and the forming of the glial scar (Mothe and Tator, 2005). This process was observed in this study through the use of GFAP and nestin double staining, however it was not quantified. Around the lesion edge, there were many GFAP and nestin double labeled cells, many of which had an astrocytic-like phenotype. Several studies have suggested that ependymal NPCs contribute to the formation of activated astrocytes and the subsequent formation of the glial scar in adult animals (Mothe and Tator, 2005; Horky et al., 2006).

Initially it was expected that the response of NPC in the infants would be significantly greater that that evident in the adult and juvenile groups. This was based on the differences between mature and infant cords described by numerous authors. As early as the 1980s Bregman and Goldberger described the “infant lesion effect” (Bregman and Goldberger, 1983a,b,c) and in 1997 Beattie et al observed greater regeneration in younger animals after a contusion SCI (Beattie et al., 1997). The greater cellular plasticity of the infant cord (Bregman and Goldberger, 1982; Brown et al., 2005), and the physiological hypermobility and malleability of the spinal cord (Kuluz et al., 2010) may contribute to this improved regeneration. Of greater interest to the current study are the levels of progenitor cell populations present in the infant CNS and also the state of continued development of the neonatal cord (Carrascal et al., 2005), which led to the expectation of a greater nestin response in the infant SCI group compared to the adult and juvenile SCI groups. This was not the case. The nestin intensity was moderately consistent between all three age groups at 24 h post injury with each age group showing a significant increase in nestin intensity around the central canal. At 1 week post injury the nestin intensity continued to increase in the two mature age groups, but the increase was not statistically different in the infant groups. All three age groups had begun to decrease by 2 weeks post injury but this was most significant in the infants which are almost back to baseline levels. It should be noted that there is currently no substantial research into the fate of NPCs in infants as compared to adults. It is possible that these cells may be differentiating to form functional neural tissue rather than the majority going toward the formation of the glial scar, contributing to the differences in lesion progression that were seen in the current study.

## Concluding remarks

There is still much to learn about SCI in young subjects and the differences in the response to the same injury between animals of different ages. There is a trend for better recovery in young animals, as well as in humans following SCI however the mechanisms behind these recovery differences remain a mystery. This study aims to start filling in some of the large gaps in our knowledge of SCI in younger animals and how the better recovery observed in these infants can be used therapeutically in mature SCI. We also aimed to develop an understanding of the interactions between cells and systems that governs the progression of SCI. To do this the response of key cellular players from the innate immune system and the CNS in SCI were examined; the first responders, neutrophils, and early responding macrophages and microglia, as well as the endogenous neural progenitor cells and resident astrocytes. The results suggested significant difference between mature animals and infants in all of the aspects examined, from the histological progression of the injury to the cellular response. The most significant differences were observed in the cellular inflammatory response, suggesting this as a key player in the observed better functional recovery in younger subjects. However, the synergy between the responses that is hinted at in this study bears much greater research and suggests that a therapeutic intervention to assist in favorable injury resolution need to focus on more than a single aspect.

## Author contributions

TS was the primary author writing and editing the article based on her honors and Ph.D. research. A large portion of the infant surgery was conducted by KM while YM conducted a large proportion of the adult surgeries and tissue processing. During the honors research many of the animals were shared between TS and TN projects. All authors contributed to the editing of this article. CG was the unit supervisor of this research and assisted in the writing of this article.

### Conflict of interest statement

The authors declare that the research was conducted in the absence of any commercial or financial relationships that could be construed as a potential conflict of interest.
